# Detecting sexually transmitted infections beyond the syndromic approach: lessons from a rural setting in Chiapas, Mexico

**DOI:** 10.3389/frph.2024.1441909

**Published:** 2024-07-24

**Authors:** Susan Gonzalez, Petra Natalia Lopez Velasco, Carlos Adolfo Mena Antonio, Daniel Palazuelos

**Affiliations:** ^1^ Pathways MD Program, Harvard Medical School, Boston, MA, United States; ^2^ Atención Primaria, Compañeros En Salud, Angel Albino Corzo, Chiapas, Mexico; ^3^ Facultad de Medicina, Universidad Nacional Autonoma de Mexico, Mexico City, Mexico; ^4^ Department of Medicine, Division of Global Health Equity, Brigham and Women’s Hospital, Boston, MA, United States

**Keywords:** sexually transmitted infection, screening, hepatitis B, Latin America, point-of-care test, global health

## Abstract

Sexually Transmitted Infections (STIs) are a critical global health concern, with low- and middle-income countries carrying the highest burden. The development of rapid point-of-care STI tests has enabled screening in settings without laboratory access. Yet, high-need settings face unique challenges that may influence the implementation and uptake of STI screening. This piece discusses lessons learned from the implementation of STI screening in a rural, low-resource setting in Chiapas, Mexico. Despite minimal privacy and a low staff-to-patient ratio, a streamlined approach was developed to destigmatize and maximize STI screening. The clinic team developed strategies through practice, including incorporating screening into triage procedures and offering screening to family members. This protocol led to an average screening rate of 37% within three months and acceptance of screening by family units. It was observed that access to treatment was necessary to alleviate patient hesitation to screening due to fears of a positive result. As STI screening increases globally, healthcare systems must develop robust access to treatment to effectively prevent and treat STIs worldwide.

## Introduction

Sexually transmitted infections (STIs) are a critical global health concern. Each day, more than one million people are infected with an STI (1). The sequelae of these infections span a range of health outcomes, from detrimental effects such as scarring and infertility to fatal ones such as ectopic pregnancies and severe infections (1). A recent study in the Lancet of over 200 countries across three decades identified that the burden of STIs is disproportionately felt by low- and middle-income countries (LMICs), with sub-Saharan Africa, Latin America, and the Caribbean having the highest age-standardized incidence of STIs (2). In response, the 2022–2030 World Health Organization (WHO) Global Health Sector Strategies for HIV, viral hepatitis, and STIs calls for prioritizing STI screening in regions and populations with the highest risk of infection and emphasizes the importance of an integrated, community-centered approach.

Historically, low-resource settings without laboratory access relied on syndromic management of STIs, yet most STIs are asymptomatic (1, 3). The development of rapid point-of-care (POC) STI tests has enabled screening in settings without laboratory access, with HIV, syphilis, and hepatitis B (Hep B) rapid tests having a higher and more reliable sensitivity and specificity than Gonorrhea and Chlamydia testing (1, 4, 5). Healthcare teams operating in low-resource settings may prefer rapid testing that is user-friendly and does not require additional parts or electricity (6, 7). Patients may also prefer the finger prick and rapid turnaround time associated with POC tests in contrast to venipuncture (6, 7).

A recent scoping review investigated the facilitators and barriers to implementing POC STI testing in LMICs (6). Eighty-two studies were included with various approaches (integration into workflow, targeted community outreach) and settings (rural, urban, clinics, homes). POC STI tests were feasible in this range of contexts and cultures, with individual and systemic factors contributing to uptake. Screening strategies that integrate with pre-existing systems can minimize disruption and thus facilitate testing (6). For example, after implementing HIV, syphilis, malaria, and anemia POC tests at antenatal health facilities in western Kenya, healthcare workers reported that through practice, they improved their skills and adapted the tests to their workflow needs (8). Individual patient characteristics, such as knowledge of STIs and trust in healthcare systems, influence testing uptake (6). One 6-country implementation study of rapid POC syphilis testing found variation in patient perspectives across settings (9). For example, the indigenous population in Brazil had concerns about a positive result while some in the Guangdong province of China declined testing due to their perceived low risk. Context-specific factors, such as transportation and cost, also impact testing uptake (6). For example, one mixed-methods analysis of rapid HIV/syphilis testing acceptance among migrant workers in China found that longer work hours hindered testing uptake (10). Of the 82 studies included in the review, 20 (24%) were conducted in Latin America, only 5 of which were in a rural setting. Of these, 3 included STI screening programs that were integrated into routine care, which involved training and collaborating with pre-established healthcare personnel (9, 11, 12).

To successfully support high-need populations, it is crucial to develop context- and population-specific screening, as these factors influence testing implementation and uptake (6). Thus, the literature needs examples of successful STI screening programs in LMICs, where healthcare systems are situated within varying ecosystems of social and sexual norms and operate with unique resource constraints (1, 6). Here we discuss lessons learned from a pilot implementation study of an STI screening program in a rural, low-resource clinic in Chiapas, Mexico.

## Context

We implemented an STI screening protocol at a community clinic located within a small rural community in Chiapas, Mexico with a population of 665. The clinic is supported by a non-governmental organization (Partners In Health, PIH) and the Mexican Secretariat of Health and functions as a primary care clinic– attending well visits, prenatal care, and chronic diagnoses management (hypertension, diabetes, epilepsy)– and attends urgent cases 24 h a day. The clinic team is comprised of one general physician, one nurse, and one community health worker (CHW), all of whom live in the community. The clinic has one private exam room, occupied by the physician and patient, and one waiting room, where the nurse and CHW triage and obtain vital signs.

Previously, the clinic's use of STI rapid testing was limited to the screening of pregnant patients during prenatal visits and the testing of symptomatic patients. However, per the WHO guidelines, screening of asymptomatic individuals who belong to at-risk populations is crucial. PIH, an organization dedicated to supporting the health of marginalized communities, took an interest in piloting STI screening for asymptomatic, general clinic patients. However, it was unclear whether clinic teams would be able to manage the added task, and it was a mystery if the patient community would accept screening.

## Program details

The clinic team carries a high workload and the success of the STI screening intervention depended on the clinical staff's buy-in (6). Therefore, to initiate, we had a collaborative conversation with the clinic staff, sharing background information regarding STIs and their consequences. Clinic team members expressed interest in screening the community– stating that they had witnessed late diagnoses of STIs and imagined screening may have helped. They began brainstorming how a protocol could be integrated into the workflow.

The STI screening program included rapid HIV, Hep B, and Syphilis tests requiring a few drops of blood, with results in minutes (Certum Diagnostics). Our rapid Gonorrhea and Chlamydia tests require a cervical or urethral sample and were excluded from this protocol due to space and time constraints. STI testing was offered during regular clinic hours in the clinic waiting room and was applied to any non-pregnant patient of reproductive age (14–49 years old) who arrived for clinic services. Pregnant patients were excluded from the protocol because they received STI screening per pre-established prenatal visit guidelines. The team acknowledged that to identify risk factors, a private patient interview is ideal, however, our clinic setting lacks privacy. Given our limitations and our desire to avoid excluding patients based on an inaccurate risk assessment, we opted to screen all non-pregnant patients of reproductive age in the waiting room so that the nurse could maximize screening. We had monthly check-ins with the team to review the percentage of patients screened and to together identify facilitators, barriers, and challenges to screening.

## Identifying risk and destigmatizing screening

The WHO recommends targeting screening for at-risk populations, wherein risk is often linked to behavior (such as sex work or drug use) or other sociodemographic factors such as migrant status, gender, and sexuality (1). However, in LMICs, the definition of “high-risk” may differ from epidemiological data in high-income countries. For example, data from high-income countries illustrates that youth are having sex earlier and people have a greater number of sexual partners over their lifetimes, in the setting of declining marriage rates (13), yet this trend may not apply in all settings. It is imperative to understand what other economic, geographic, and sociocultural dynamics may influence risk. For example, in Mexico and other countries, marriage is a risk factor for HIV infection in women (14). One study in Mexico City found that the majority of HIV infections in women were from a stable male partner (15). Interviews with women in a rural town in Mexico revealed that sociocultural factors, including the value of marriage and fidelity, and men's international migration for labor opportunities, posed a risk for HIV infection in women with stable male partners (14). We witness similar patterns in our context: in general, women do not leave the community as often as their male partners, who travel often to sell their crops and coffee; during such travel, men may engage in extramarital sex. This sociocultural dynamic illustrates how screening for typical risk factors is insufficient in our context: although low-risk per WHO guidelines, we identified a married, 31-year-old woman with a reactive rapid blood test for Hep B. This case exemplifies the value of expanding the definition of risk to fit the unique qualities of a community and inclusively screen.

We adopted the following strategies to maximize STI screening: (1) Offer the testing as part of the patient's vital signs. Combining the testing with typical clinic processes helped normalize the testing. (2) If a patient requires a fingerpick (i.e., for rapid blood glucose or hemoglobin tests), conduct the rapid STI tests in succession to minimize the number of fingerpicks per patient and maximize workflow. (3) Offer the testing to all eligible family members accompanying the patient. Offering the testing broadly helped illustrate that we were not offering screening based on individual qualities. In a setting where the family unit is valued highly, we saw 100% acceptance of testing by accompanying family members if the patient accepted the testing. Altogether, these strategies helped us to reframe STI testing as a necessary and normal part of the clinic visit for any eligible patient. Despite previously screening an average of 0% of the general clinic population, within three months, the clinic team achieved an average screening rate of 37% of eligible, non-symptomatic, non-pregnant patients between the ages of 14–49.

## Discussion: prevention beyond STI screening

Overall, our pilot STI screening project was successfully integrated into the clinic workflow and saw high acceptance from the patient community, with almost 40% of eligible patients receiving screening in the first three months. Due to time constraints, urgent care visits, and cross-coverage duties, screening was not offered to all eligible patients. Some patients declined testing due to the lack of privacy, stating they would return when the clinic was less busy. Other patients declined due to fear of a positive result, stating that they would rather not know their results if treatment were not accessible. If treatment is available, accessible, and affordable, patients may be more likely to accept testing (16). This reflects lessons from the HIV epidemic: across international, high-prevalence settings, it was found that access to treatment and resources protected against HIV-related stigma and discrimination (17). Luckily, PIH has the funding to support treatment (antivirals, antibiotics), referrals, and transportation if needed. Hesitant patients were assured that if positive, they would be connected to treatment, and many subsequently accepted screening. Patients with reactive tests expressed a desire for treatment and brought their family members to the clinic to be tested, suggesting acceptance.

Our project was rooted in partnership with the clinic staff; we prioritized clinic team buy-in and encouraged them to design an approach that worked within their workflow and space. Through experience, the team astutely identified strategies to maximize screening. To initiate a broad STI screening strategy, we recommend collaborating with pre-existing systems to avoid disruption and increase sustainability. For example, implementers can ask the team when and where screening activities would be most feasible. Frequent team check-ins allow for positive feedback and troubleshooting, which increase morale and testing uptake. As we saw, rapid POC STI tests have enabled screening in low-resource settings with high disease burden, yet screening programs must be paired with robust support for treatment to decrease stigma and maximize screening. To address STIs, a comprehensive healthcare system would include screening, vaccination where applicable (Hep B), affordable testing, and accessible medical treatment (i.e., low medication costs, transportation support). One example of a successful, comprehensive program is Iran's Triangular Clinic, which manages patients with HIV, STIs and addiction (18). The clinic not only screens for and treats these conditions, but also offers comprehensive services such as tuberculosis screening, community engagement programs (i.e., summer camps, outdoor activities), and primary care. Yet, challenges and inequities persist. For example, despite incredible advances in Hep B vaccination globally, there are still global disparities. LMIC countries have lower vaccine coverage and low-resource and remote locations face barriers such as vaccine availability and transportation (19).

Overall, our experience taught us valuable lessons regarding strategies to maximize STI screening in a rural, low-resource setting in Latin America, including expanding the definition of “at-risk” to screen inclusively and offering screening to all family members. However, our approach was facilitated by our access to rapid STI tests. In reality, infrastructure issues such as stockouts and prices are a barrier for many (6). As STI screening increases globally, there must also be enthusiasm and funding for robust health systems to adequately treat diagnosed patients and prevent the spread of disease.

## Data Availability

The raw data supporting the conclusions of this article will be made available by the authors, without undue reservation.
